# *C*. *elegans* RAB-35: Dual roles in apoptotic cell clearance

**DOI:** 10.1371/journal.pgen.1007534

**Published:** 2018-08-23

**Authors:** Christian E. Rocheleau

**Affiliations:** 1 Division of Endocrinology and Metabolism, Department of Medicine, McGill University, Montreal, Quebec, Canada; 2 Department of Anatomy and Cell Biology, McGill University, Montreal, Quebec, Canada; 3 Program in Metabolic Disorders and Complications, Research Institute of the McGill University Health Centre, Montreal, Quebec, Canada; NYU School of Medicine, UNITED STATES

The phagocytic clearance of apoptotic cells is important for maintaining tissue homeostasis, and defects in phagocytic clearance can lead to inflammatory diseases and autoimmunity [1]. While much is known about how apoptotic cells are cleared, there are many gaps in our knowledge. In this issue, Zheng Zhou and colleagues at Baylor College of Medicine report the identification of Rab35 as a new regulator of apoptotic cell clearance using the nematode *Caenorhabditis elegans* [2].

*C*. *elegans* is an attractive model for the in vivo study of apoptotic cell clearance. During development, 131 somatic cells undergo apoptosis in an invariant manner and in the adult germline approximately 50% of germ cells die by apoptosis [3, 4]. In both cases, apoptotic cells are quickly engulfed by neighboring cells and degraded. Genetic screens for persistent apoptotic cells have identified genes and pathways that mediate apoptotic cell recognition, phagocytic engulfment, and phagosome maturation and degradation [3–5]. Two parallel genetic pathways regulate apoptotic cell engulfment [3]. Cell death abnormality (CED)-1, a scavenger receptor, functions with CED-6 engulfment adaptor PTB domain containing 1 (Gulp1) and the CED-7 adenosine triphosphate-binding cassette (ABC) transporter to recognize phosphatidylserine on dying cells. The other pathway, defined by the CED-2 adaptor protein, the CED-10 Rac1 guanosine triphosphate hydrolase (GTPase), and its bipartite guanine nucleotide exchange factor (GEF) CED-5 and CED-12, regulates actin polymerization. Loss of both pathways does not completely block apoptotic cell clearance [6], suggesting that there may be additional players.

Once internalized, apoptotic cell-containing phagosomes undergo maturation. Phosphatidylinositol 4,5-phosphate (PI[4,5]P) is replaced with PI(3)P [7]. This replacement is mediated in part by the loss of the myotubularin (MTM)-1 PI3-phosphatase, a PI(4,5)P effector, and the activity of the class II and class III PI3-kinases, phosphoinositide-3-kinase (PIKI)-1, and vacuolar protein sorting (VPS)-34, permitting the recruitment of PI(3)P binding proteins such as the sorting nexin (SNX)-1 [7–9]. Several Rab GTPases control phagosome maturation and lysosomal degradation. During maturation, RAB-5 recruits the SAND-1/CCZ-1 GEF, which in turn recruits and activates RAB-7 [10, 11], which along with RAB-2/uncoordinated (UNC)-108 and RAB-14 promote fusion with lysosomes [12]. In an RNA-mediated interference (RNAi) screen for additional Rab GTPases that mediate apoptotic cell clearance, Haley and colleagues identify RAB-35 as a novel regulator of apoptotic cell clearance [2]. *C*. *elegans* RAB-35 was previously found to regulate endosome recycling of the receptor-mediated endocytosis (RME)-2 yolk receptor in oocytes [13]. In cells from other organisms, Rab35 has been implicated in regulation of the actin cytoskeleton and phagocytosis via regulation of the cell division cycle 42 (Cdc42), Rac1, and ADP-ribosylation factor 6 (Arf6) GTPases [14–16].

Haley and colleagues describe a novel in vivo role for the RAB-35 GTPase in the clearance of apoptotic cells in *C*. *elegans* [2]. The authors show that *rab-35* mutants have persistent apoptotic cells and that *rab-35* is required in the engulfing cell, consistent with a defect in phagocytosis and/or phagosome maturation. They find that RAB-35 must cycle between its on and off states to function, and its localization to phagosomes is increased in the on state. They link RAB-35 activation in phagocytosis to FLCN-1 (Folliculin1), a candidate RAB-35 GEF distinct from RME-4, the RAB-35 GEF in yolk trafficking, providing important in vivo evidence to support the biochemical data with the mammalian homologs [17]. The authors also found evidence for Tre-2/Bub2/Cdc16 (TBC-10) as the relevant RAB-35 GTPase activating protein (GAP) during phagocytosis, consistent with TBC1D10 functioning as a Rab35 GAP in mammalian cells [18] (Fig 1).

**Fig 1:**
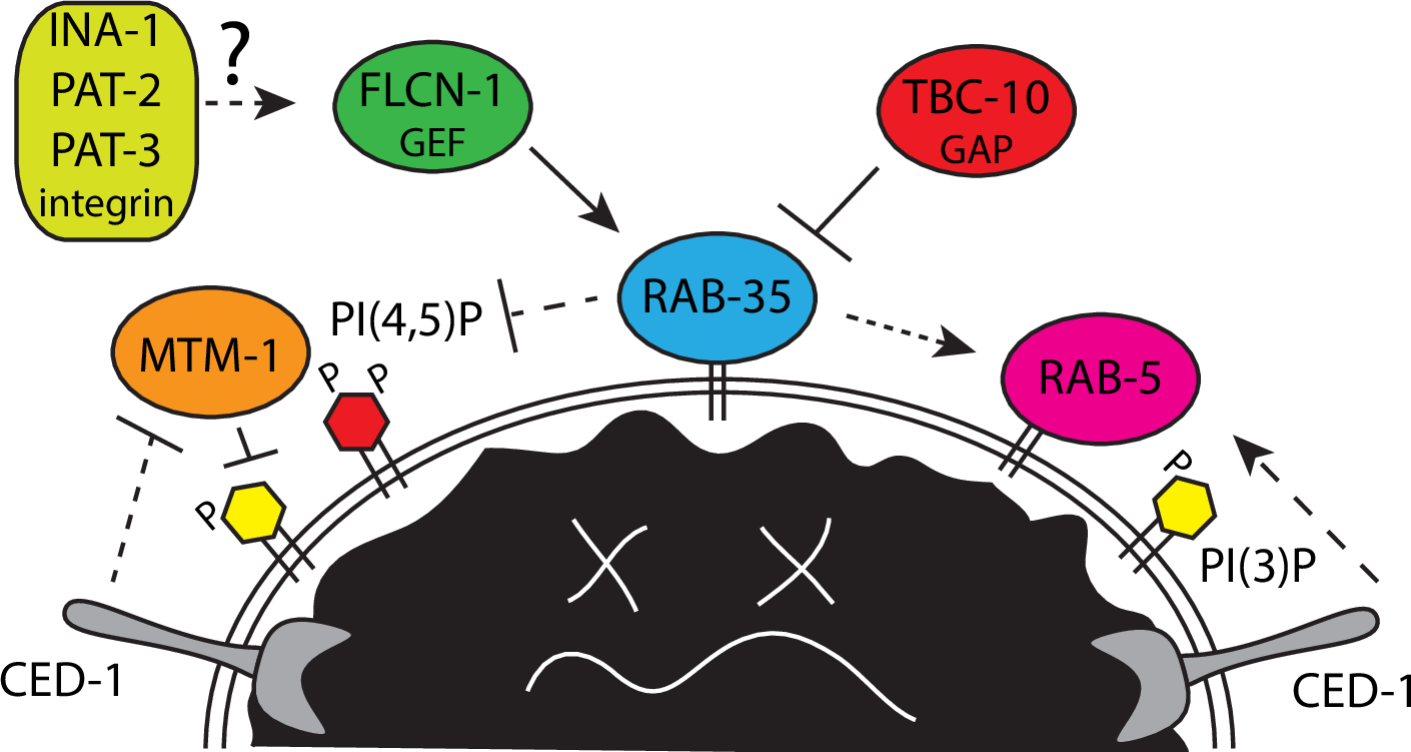
Model of RAB-35 regulation and function during early phagosome maturation. FLCN-1 activates RAB-35 downstream of an unknown signal, possibly integrin, promoting phagosome localization. RAB-35 functions parallel to CED-1 to promote the removal of PI(4,5)P and MTM-1, thus permitting the accumulation of PI(3)P. RAB-35, CED-1, and PI(3)P may promote RAB-5 recruitment and activation on maturing phagosomes. The RAB-35 effector(s) that mediate these events are not yet known and are thus depicted by dashed lines. RAB-35 would then be inactivated by TBC-10, which theoretically could be recruited by RAB-5 or other factors on the maturing phagosome.

RAB-35 localizes to developing pseudopods and has increased localization on phagosomes, suggesting a function in early phagocytosis events that correlate with the loss of PI(4,5)P and the gain of PI(3)P [2]. In *rab-35* mutants, PI(4,5)P and MTM-1 persists on phagosomes and the accumulation of PI(3)P and PI(3)P binding proteins are delayed. Also delayed is the recruitment of RAB-5, the first in a series of Rab GTPases required for efficient phagosome maturation. Genetic epistasis, together with the finding that RAB-35 localization to phagosomes precedes RAB-5, suggests that RAB-35 functions upstream of RAB-5 in a common pathway.

The CED-1 receptor also regulates early steps of phagosome maturation in addition to its role in apoptotic cell recognition and engulfment [2, 19]. Although *ced-1* and *rab-35* are phenotypically similar, *ced-1; rab-35* double mutants displayed more severe maturation defects, including delayed accumulation of PI(3)P and RAB-5 on phagosome membranes, suggesting that they function in parallel pathways [2] (Fig 1).

Haley and colleagues identified a second role for RAB-35 in apoptotic cell recognition [2]. They find that *rab-35* mutants show a delay in apoptotic cell recognition. *rab-35* mutants strongly enhance the apoptotic cell corpse recognition phenotypes of both *ced-1* and *ced-5* mutants, and this is more severe in a *ced-1; rab-35; ced-5* triple null mutant. The authors conclude that *rab-35* functions in a third pathway in parallel to the *ced-1*/*-6*/*-7* and *ced-2*/*-5*/*-10*/*-12* pathways.

Integrin has been proposed to function as an apoptotic cell receptor in *C*. *elegans* upstream of SRC-1 tyrosine kinase, CDC-42, and the CED-10 pathway [20, 21, 22]. Haley and colleagues confirmed that RNAi of either of the two *C*. *elegans* integrin α subunits *(ina-1* and *pat-2*) or the single β subunit (*pat-3*) results in an apoptotic cell clearance phenotype. Genetic epistasis indicates that the integrins function in the same pathway as *rab-35*.

Overall, these findings represent a significant advance in the field, defining dual roles for RAB-35 in apoptotic cell clearance in both apoptotic cell recognition and early phagosome maturation. With new knowledge comes new questions. What are the functional relationships between integrin and RAB-35? Integrin signaling could potentially activate RAB-35 via recruitment of FLCN-1 (Fig 1). Alternatively, RAB-35 might regulate endosome recycling of integrin as it does the RME-2 yolk receptor. Determining if integrin regulates FLCN-1 and RAB-35 localization and if RAB-35 regulates integrin localization may shed some light on the mechanisms involved.

During early phagosome maturation, RAB-35 regulates the transition of PI(4,5)P to PI(3)P and the recruitment of RAB-5 [2]. These novel roles of RAB-35 are likely carried out by one or more effector proteins. The authors note PI4- and PI5-kinases and phosphatases as good candidates. In mammals, oculocerebrorenal syndrome of Lowe (OCRL), an inositol 5-phosphatase, is a Rab35 effector that mediates PI(4,5)P breakdown [23]. Especially appealing is that loss of *C*. *elegans ocrl-1* results in persistent cell corpses, persistent PI(4,5)P on phagosomes, as well as a delay in RAB-5 recruitment [7]. Therefore, future analysis should determine if OCRL-1 is recruited and regulated by RAB-35 during phagosome maturation. While the recruitment of RAB-5 by RAB-35 could be indirect via regulation of PIPs, it is interesting to speculate a more direct regulation through a Rab cascade as seen with RAB-5 and RAB-7 [11, 24]. In this scenario, RAB-35 could recruit a RAB-5 GEF to activate RAB-5, while RAB-5 could recruit the TBC-10 GAP to inactivate RAB-35. Finally, it would be interesting to determine if RAB-35 might regulate CED-10, CDC-42, or ARF-6 activity, as suggested from *Drosophila* and mammalian cell culture studies [15, 16]. This work will surely fuel more discoveries and continue to delineate the pathways regulating apoptotic cell clearance.

## References

[1] Poon IK, Lucas CD, Rossi AG, Ravichandran KS. Apoptotic cell clearance: basic biology and therapeutic potential. Nat Rev Immunol. 2014;14(3):166–80. Epub 2014/02/01. 10.1038/nri3607 ; PubMed Central PMCID: PMCPMC4040260. 24481336 PMC4040260

[2] Haley RC, Wang Y, Zhou Z. The small GTPase RAB-35 leads a third pathway to aid the recognition and 3 degradation of apoptotic cells. PLoS Genet. 2018. 10.1371/journal.pgen.1007558 PMC6107108 30138370

[3] Wang X, Yang C. Programmed cell death and clearance of cell corpses in Caenorhabditis elegans. Cell Mol Life Sci. 2016;73(11–12):2221–36. Epub 2016/04/07. 10.1007/s00018-016-2196-z . 27048817 PMC11108496

[4] Malin JZ, Shaham S. Cell Death in C. elegans Development. Curr Top Dev Biol. 2015;114:1–42. Epub 2015/10/04. 10.1016/bs.ctdb.2015.07.018 ; PubMed Central PMCID: PMCPMC5206663. 26431562 PMC5206663

[5] Ellis RE, Jacobson DM, Horvitz HR. Genes required for the engulfment of cell corpses during programmed cell death in Caenorhabditis elegans. Genetics. 1991;129(1):79–94. Epub 1991/09/01. ; PubMed Central PMCID: PMCPMC1204584. 1936965 10.1093/genetics/129.1.79 PMC1204584

[6] Yu X, Odera S, Chuang CH, Lu N, Zhou Z. C. elegans Dynamin mediates the signaling of phagocytic receptor CED-1 for the engulfment and degradation of apoptotic cells. Dev Cell. 2006;10(6):743–57. Epub 2006/06/03. 10.1016/j.devcel.2006.04.007 . 16740477

[7] Cheng S, Wang K, Zou W, Miao R, Huang Y, Wang H, et al PtdIns(4,5)P(2) and PtdIns3P coordinate to regulate phagosomal sealing for apoptotic cell clearance. J Cell Biol. 2015;210(3):485–502. Epub 2015/08/05. 10.1083/jcb.201501038 ; PubMed Central PMCID: PMCPMC4523610. 26240185 PMC4523610

[8] Lu N, Shen Q, Mahoney TR, Neukomm LJ, Wang Y, Zhou Z. Two PI 3-kinases and one PI 3-phosphatase together establish the cyclic waves of phagosomal PtdIns(3)P critical for the degradation of apoptotic cells. PLoS Biol. 2012;10(1):e1001245 10.1371/journal.pbio.1001245 ; PubMed Central PMCID: PMC3260314. 22272187 PMC3260314

[9] Lu N, Shen Q, Mahoney TR, Liu X, Zhou Z. Three sorting nexins drive the degradation of apoptotic cells in response to PtdIns(3)P signaling. Mol Biol Cell. 2011;22(3):354–74. Epub 2010/12/15. 10.1091/mbc.E10-09-0756 ; PubMed Central PMCID: PMCPMC3031466. 21148288 PMC3031466

[10] Kinchen JM, Doukoumetzidis K, Almendinger J, Stergiou L, Tosello-Trampont A, Sifri CD, et al A pathway for phagosome maturation during engulfment of apoptotic cells. Nat Cell Biol. 2008;10(5):556–66. 10.1038/ncb1718 . 18425118 PMC2851549

[11] Kinchen JM, Ravichandran KS. Identification of two evolutionarily conserved genes regulating processing of engulfed apoptotic cells. Nature. 2010;464(7289):778–82. 10.1038/nature08853 . 20305638 PMC2901565

[12] Guo P, Wang X. Rab GTPases act in sequential steps to regulate phagolysosome formation. Small GTPases. 2010;1(3):170–3. Epub 2011/06/21. 10.4161/sgtp.1.3.14511 ; PubMed Central PMCID: PMCPMC3116604. 21686272 PMC3116604

[13] Sato M, Sato K, Liou W, Pant S, Harada A, Grant BD. Regulation of endocytic recycling by C. elegans Rab35 and its regulator RME-4, a coated-pit protein. Embo J. 2008;27(8):1183–96. 10.1038/emboj.2008.54 . 18354496 PMC2367398

[14] Zhang J, Fonovic M, Suyama K, Bogyo M, Scott MP. Rab35 controls actin bundling by recruiting fascin as an effector protein. Science. 2009;325(5945):1250–4. Epub 2009/09/05. 10.1126/science.1174921 . 19729655

[15] Shim J, Lee SM, Lee MS, Yoon J, Kweon HS, Kim YJ. Rab35 mediates transport of Cdc42 and Rac1 to the plasma membrane during phagocytosis. Mol Cell Biol. 2010;30(6):1421–33. Epub 2010/01/13. 10.1128/MCB.01463-09 ; PubMed Central PMCID: PMCPMC2832496. 20065041 PMC2832496

[16] Egami Y, Fukuda M, Araki N. Rab35 regulates phagosome formation through recruitment of ACAP2 in macrophages during FcgammaR-mediated phagocytosis. J Cell Sci. 2011;124(Pt 21):3557–67. Epub 2011/11/03. 10.1242/jcs.083881 . 22045739

[17] Nookala RK, Langemeyer L, Pacitto A, Ochoa-Montano B, Donaldson JC, Blaszczyk BK, et al Crystal structure of folliculin reveals a hidDENN function in genetically inherited renal cancer. Open Biol. 2012;2(8):120071 Epub 2012/09/15. 10.1098/rsob.120071 ; PubMed Central PMCID: PMCPMC3438538. 22977732 PMC3438538

[18] Chaineau M, Ioannou MS, McPherson PS. Rab35: GEFs, GAPs and effectors. Traffic. 2013;14(11):1109–17. Epub 2013/08/03. 10.1111/tra.12096 . 23905989

[19] Yu X, Lu N, Zhou Z. Phagocytic receptor CED-1 initiates a signaling pathway for degrading engulfed apoptotic cells. PLoS Biol. 2008;6(3):e61 10.1371/journal.pbio.0060061 . 18351800 PMC2267821

[20] Hsieh HH, Hsu TY, Jiang HS, Wu YC. Integrin alpha PAT-2/CDC-42 signaling is required for muscle-mediated clearance of apoptotic cells in Caenorhabditis elegans. PLoS Genet. 2012;8(5):e1002663 Epub 2012/05/23. 10.1371/journal.pgen.1002663 ; PubMed Central PMCID: PMCPMC3355063. 22615577 PMC3355063

[21] Neukomm LJ, Zeng S, Frei AP, Huegli PA, Hengartner MO. Small GTPase CDC-42 promotes apoptotic cell corpse clearance in response to PAT-2 and CED-1 in C. elegans. Cell death and differentiation. 2014;21(6):845–53. Epub 2014/03/19. 10.1038/cdd.2014.23 ; PubMed Central PMCID: PMCPMC4013519. 24632947 PMC4013519

[22] Hsu TY, Wu YC. Engulfment of apoptotic cells in C. elegans is mediated by integrin alpha/SRC signaling. Curr Biol. 2010;20(6):477–86. Epub 2010/03/17. 10.1016/j.cub.2010.01.062 . 20226672

[23] Dambournet D, Machicoane M, Chesneau L, Sachse M, Rocancourt M, El Marjou A, et al Rab35 GTPase and OCRL phosphatase remodel lipids and F-actin for successful cytokinesis. Nat Cell Biol. 2011;13(8):981–8. Epub 2011/06/28. 10.1038/ncb2279 . 21706022

[24] Poteryaev D, Datta S, Ackema K, Zerial M, Spang A. Identification of the switch in early-to-late endosome transition. Cell. 2010;141(3):497–508. 10.1016/j.cell.2010.03.011 . 20434987

